# Validation and development of a new automatic algorithm for time resolved segmentation of the left ventricle in magnetic resonance imaging

**DOI:** 10.1186/1532-429X-17-S1-P68

**Published:** 2015-02-03

**Authors:** Jane Tufvesson, Erik Hedström, Katarina Steding-Ehrenborg, Marcus Carlsson, Håkan Arheden, Einar Heiberg

**Affiliations:** 1 Cardiac MR group Lund, Dept. of Clinical Physiology, Lund University, Lund, Sweden; 2 Dept of Numerical Analysis, Faculty of Engineering, Lund University, Lund, Sweden; 3 Dept of Diagnostic Radiology, Lund University, Lund, Sweden

## Background

Manually delineating the left ventricle (LV) is considered the clinical standard for quantification of cardiovascular magnetic resonance (CMR) images, despite being time consuming and observer dependent. Previous automatic methods generally do not account for the long-axis motion, which is a major contributor to the stroke volume. Therefore, the aim of this study was to develop and validate an automatic algorithm for time-resolved segmentation covering the whole LV, including basal slices moving out of the imaging plane during systole.

## Methods

Ninety subjects were imaged using a routinely applied cine balanced steady state free precession (bSSFP) sequence (training set n=40, test set n=50).

The automatic algorithm uses deformable model with Expectation-Maximization (EM), followed by automatic removal of papillary muscles and detection of the outflow tract.

The reference standard was manual delineation and second observer analysis was performed in a subset of patients (n=25).

## Results

**Figure 1:**
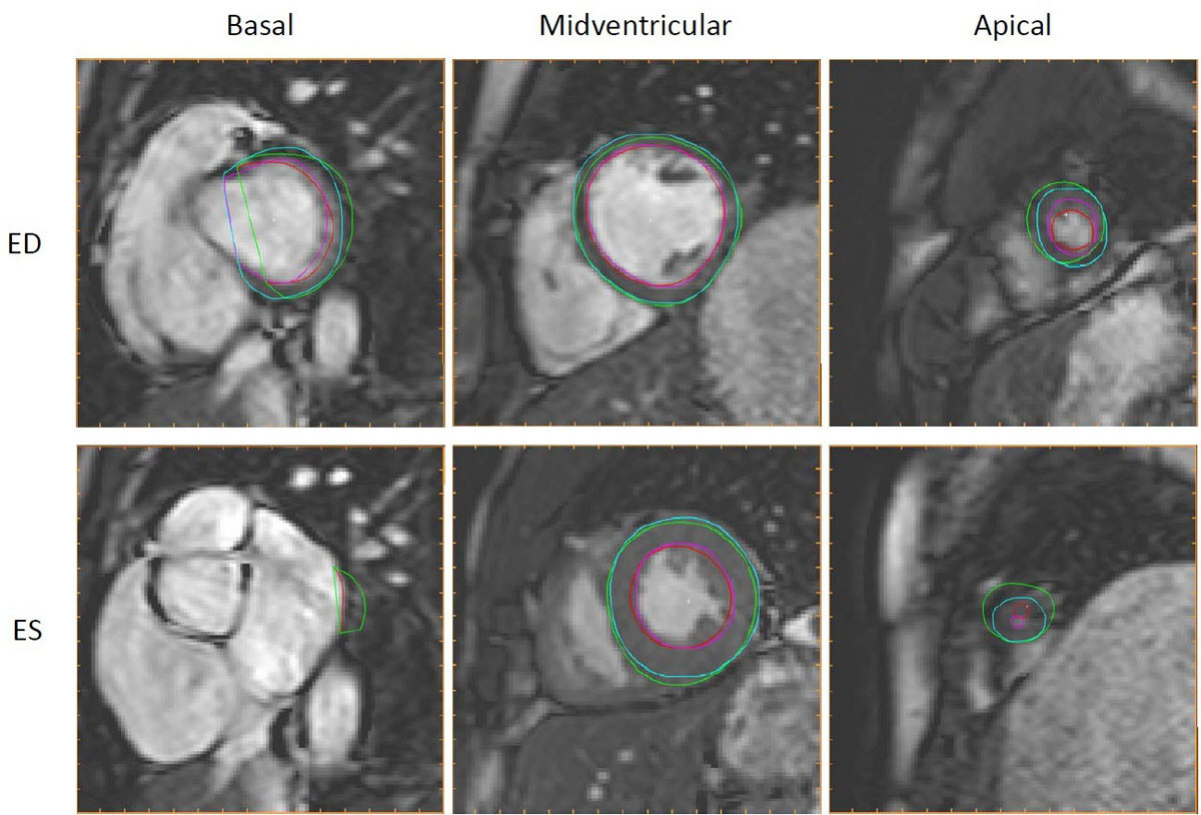
**Automatic segmentation compared to manual delineation in basal, midventricular and apical slices.** Automatic segmentation (endocardium in red, epicardium in green) and manual delineation (endocardium in pink, epicardium in light blue) shown in end diastole (ED, top row) and end systole (ES, bottom row) for the most basal slice with outflow tract moving out of the imaging plane during systole (left column), a midventricular slice including papillaries (middle column) and an apical slice with minimal lumen in end systole (right column).

**Figure 2:**
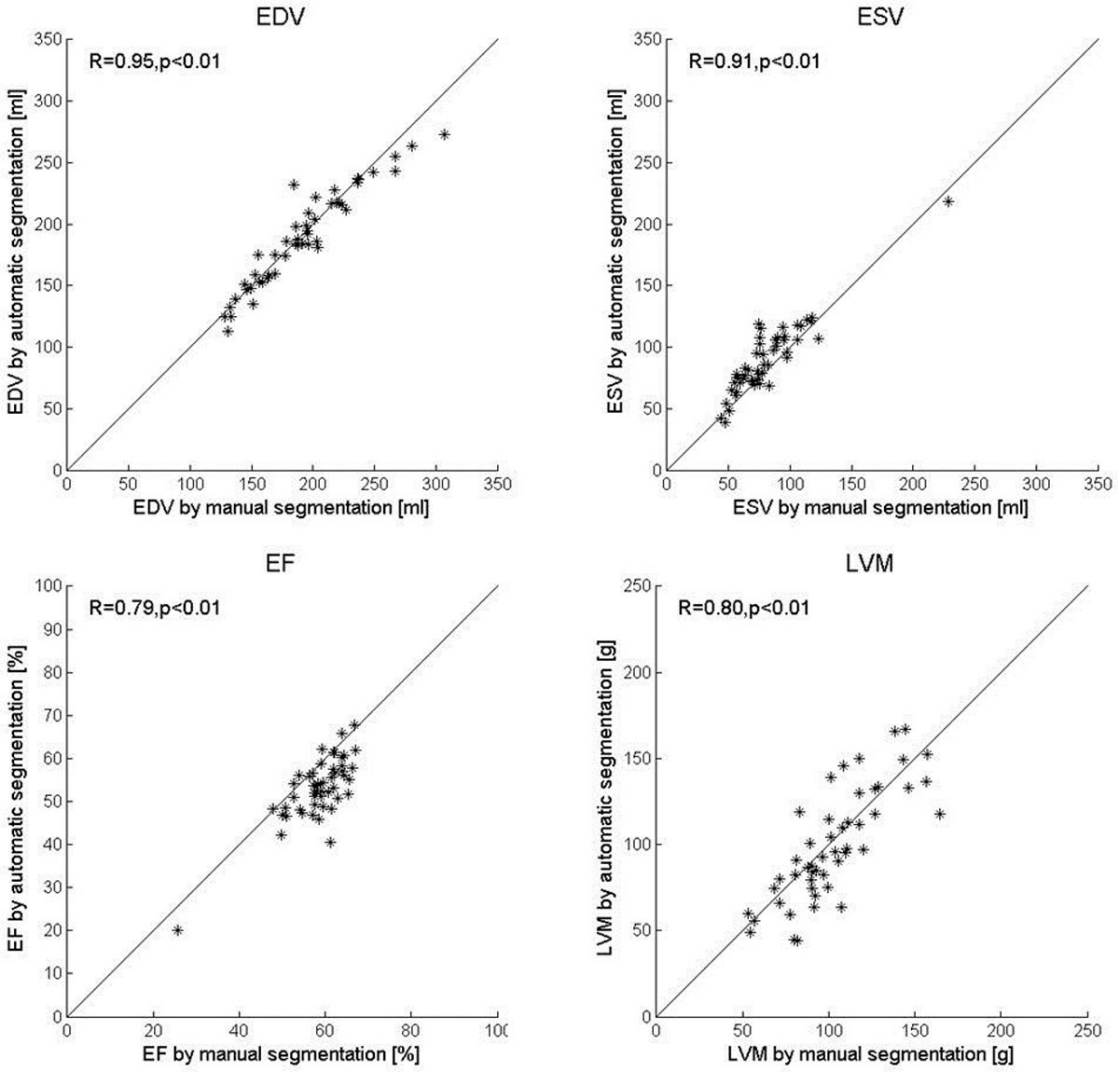
**Correlations between automatic segmentation and manual delineation.** Automatic segmentation plotted against manual delineation for end-diastolic volume (EDV, top left), end-systolic volume (ESV, top right), ejection fraction (EF, bottom left) and left ventricular mass (LVM, bottom right). Lines indicate the line of identity.

Typical automatic segmentation and reference manual delineation is shown in three selected slices in Figure 1. The differences between automatic segmentation and reference manual delineation were EDV -2.4±12.8 ml, ESV 9.1±12.3 ml, EF -5.4±4.7 % and LVM -3.5±19.5 g (Figure 2, n=49 after exclusion due to severe artefacts in 1 patient). In the second observer sub set differences between second observer manual delineation and reference manual delineation were EDV 10.5 ± 4.4 ml, ESV 5.1 ± 4.7 ml, EF -0.4 ± 2.3 % and LVM -7.4 ± 8.9 g compared to the differences between automatic segmentation and the reference manual delineation which were EDV -1.0 ± 9.6 ml, ESV 9.2 ± 10.0, EF -4.9 ± 4.0 % and LVM -10.5 ± 17.5 g.

## Conclusions

The proposed automatic LV segmentation algorithm reached accuracy comparable to that found between observers, taking automatic segmentation one step closer to clinical routine. The algorithm and all images with manual delineations will be made available for benchmarking.

## Funding

This study has been funded by the Swedish Research Council, The Swedish Heart and Lung Foundation, The Medical Faculty of Lund University, Sweden, and Region of Scania, Sweden.

